# A Practical Review of Cytomegalovirus in Gastroenterology and Hepatology

**DOI:** 10.1155/2019/6156581

**Published:** 2019-03-07

**Authors:** Ali Y. Fakhreddine, Catherine T. Frenette, Gauree G. Konijeti

**Affiliations:** ^1^Division of Gastroenterology and Hepatology, Scripps Clinic, La Jolla, CA, USA; ^2^Division of Organ Transplantation, Scripps Green Hospital and Scripps Clinic, La Jolla, CA, USA

## Abstract

Human cytomegalovirus (CMV) is a ubiquitous *Herpesviridae* virus with a wide spectrum of pathology in humans. Host immunity is a major determinant of the clinical manifestation of CMV and can vary widely in the gastroenterology and hepatology practice setting. Immunocompetent patients generally develop a benign, self-limited mononucleosis-like syndrome whereas gastrointestinal tissue-invasive disease is more frequently seen in immunocompromised and inflammatory bowel disease patients. Additionally, liver allograft dysfunction is a significant consequence of CMV infection in liver transplant patients. While polymerase chain reaction and immunohistochemistry techniques allow for the reliable and accurate detection of CMV in the human host, the diagnostic value of different serologic, endoscopic, and histologic tests depends on a variety of factors. Similarly, latent CMV, CMV infection, and CMV disease carry different significance depending on the patient population, and the decision to initiate antiviral therapy can be complex and patient-specific. This review will focus on the pathophysiology, diagnosis, and management of CMV in patient populations relevant to the practice of gastroenterology and hepatology—liver transplant recipients, inflammatory bowel disease patients, and otherwise immunocompetent patients.

## 1. Introduction

Human cytomegalovirus (CMV) earns its name from the characteristic cytomegalic appearance of intranuclear inclusions in infected cells, an appearance first described in 1881 [1]. As a member of the *Herpesviridae* family, human herpes virus 5 (HHV 5), or CMV, is a double-stranded DNA virus capable of a wide spectrum of disease in humans. This review will focus on the diagnosis and management of CMV in the general population, liver transplant (LT) patients, and inflammatory bowel disease (IBD) patients.

## 2. Transmission and Infection

Transmission among adults can occur via exposure to bodily fluids including tears, saliva, semen, and blood or transplanted organ tissue. The main routes are via close contact with young children and intimate contact with adults such as kissing or sexual intercourse. Successful transmission is based on the frequency of these events and the chance of active viral shedding in the infected host [2].

Initial infection occurs in mucosal epithelial cells, and viral dissemination occurs via infected circulating CD14+ monocytes [3]. CMV has a remarkable doubling time of approximately one day, and clinical manifestations of infection increase proportionately to viral load [4]. In immunocompetent individuals, an initial immune response will result in controlling further replication and dissemination of virus, but a subsequent latent phase is universally present [5].

While human CMV is highly species-specific, it demonstrates broad tropism within the human body that includes parenchymal, connective tissue, and hematopoietic cells. In the liver, CMV most frequently infects hepatocytes and macrophages, whereas stromal and vascular endothelial cells are the primary target in the gastrointestinal (GI) tract [6–8]. Although smooth muscle and epithelial cells are also infected in the GI tract, inclusion bodies are rarely seen in epithelial cells around ulcer margins, supporting the common teaching of targeting the ulcer base during endoscopic biopsy of suspected CMV disease [9, 10].

Given the protean nature of CMV clinical disease following exposure, a set of universal terms is used to describe CMV pathology within the human host [11]. *Latent CMV* refers to presence of CMV viral DNA within the human host without detectable, active replication. *CMV infection* refers to evidence of active viral replication without symptoms, and *CMV disease* refers to CMV infection with overt symptoms.

## 3. CMV in the General Population: Presentation, Diagnosis, and Management

A minority of primary CMV infections in immunocompetent patients will result in symptoms. In such cases, a mononucleosis-like picture is seen with a variable degree of constitutional and organ-specific symptoms. In the general population, more severe tissue-invasive disease of the gastrointestinal tract or liver is almost always limited to patients with critical illness or comorbidities conferring relative immunosuppression.

### 3.1. Latent CMV

By far, the most common clinical status of CMV in humans is latent infection. Latent CMV is asymptomatic and diagnosed based on presence of CMV-specific IgG antibodies. CMV seroprevalence in the United States is reported to be 42-93% [2]. Female gender, older age, non-Hispanic black and Mexican American ethnicity, crowding, low education level, and low household income were all independently associated with CMV seropositivity (Table 1) [2]. The role of latent CMV in the development of GI disease is limited. CMV has been demonstrated to preferentially replicate within dysplastic colonic epithelial cells, with effects of viral proteins on various tumor suppression genes such as Bcl-2 and p53. Causality of latent CMV in GI malignancy remains implied but has not yet been demonstrated [12]. No curative treatments for latent CMV exist currently.

### 3.2. Mononucleosis-Like Syndrome

The typical presentation of mononucleosis-like syndrome consists of prolonged fevers, myalgia, and malaise. Less than 5% of patients present with jaundice. Atypical leukocytes are almost always seen. The liver is a primary site of involvement with 70-90% of patients presenting with atypical liver chemistries and 10-38% of patients having hepatosplenomegaly [13]. The ALT, AST, total bilirubin, and alkaline phosphatase are usually elevated within 3x upper limit of normal (ULN) and rarely 5x ULN. A mixed hepatocellular and cholestatic picture is generally seen [14].

**Figure 1:**
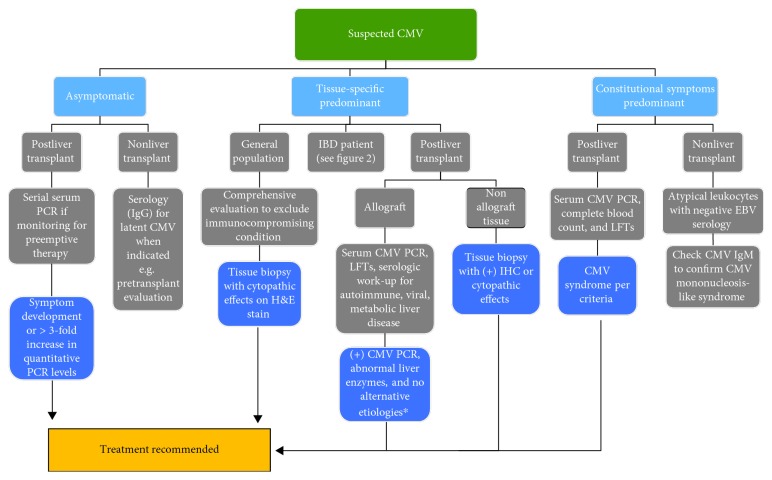
Flow diagram for diagnostic and management approach to patients with suspected CMV. ^∗^Consider liver biopsy to rule out acute graft rejection. LFT: liver function tests.

Diagnosis of mononucleosis-like syndrome due to CMV should begin with exclusion of Epstein-Barr virus (EBV) mononucleosis. Proposed diagnostic algorithms suggest testing with CMV IgM only in patients with atypical leukocytes and negative EBV heterophile antibody and EBV IgM (Figure 1). Polymerase chain reaction- (PCR-) based tests are not routinely recommended due to cost, potential for detection of latent CMV, and the adequate sensitivity and specificity of serology [15, 16].

Mononucleosis-like syndrome in immunocompetent patients is generally benign and self-limited. Although no high-quality studies exist regarding antiviral treatment of CMV mononucleosis-like syndrome, antiviral treatment for EBV mononucleosis, with or without steroids, has not demonstrated efficacy in clinically important outcomes [17, 18]. Effective treatment with ganciclovir for severe CMV mononucleosis-like syndrome has been reported. However, in the absence of a controlled study, the true benefit of antiviral therapy over observation cannot be determined [19].

### 3.3. Tissue-Invasive Disease

#### 3.3.1. Gastrointestinal

Tissue-invasive gastrointestinal (TI-GI) CMV is defined as CMV disease with symptoms localized to the GI tract. In the general population, TI-GI CMV disease almost always occurs in patients with relative immunosuppression due to critical illness or comorbidities such as type 2 diabetes mellitus, renal insufficiency, pregnancy, autoimmune diseases, heart failure, or malignancy [20–22].

In a systematic review of tissue-invasive CMV in patients with relative immunosuppression, the GI tract is most commonly involved and comprises 30% of tissue-invasive disease [21]. In all patients undergoing endoscopy at one center, approximately 30% of TI-GI CMV cases occurred in patients without overtly compromised immunity or IBD, with an overall prevalence of approximately 3 in 1,000 endoscopies [10]. Among TI-GI CMV in this population, the colon is the most frequently reported site of involvement, comprising up to 94% of cases [10, 23, 24].

Critical illness is a major risk factor for viral reactivation in otherwise immunocompetent patients, with approximately 3-4% of patients in the intensive care unit developing tissue-invasive CMV disease [25]. Active malignancy, especially hematologic malignancy when associated with low BMI and lymphopenia, is another particularly susceptible population. Blood transfusion is a well-identified risk factor among these two groups of patients (Table 1) [26, 27].

Clinical manifestations of TI-GI CMV vary and depend largely on site of involvement. Odynophagia occurs in over 60% of patients with esophageal involvement, whereas epigastric pain and hematochezia are more characteristic of gastric and colonic involvement, respectively [9]. Symptoms of fever, anorexia, weight loss, abdominal pain, nausea, vomiting, diarrhea, and hemorrhage can otherwise be present with involvement of any part of the GI tract. Endoscopic findings also range from mucosal inflammation and edema to pseudotumors and severe ulceration [9, 24, 28].

#### 3.3.2. Hepatic

Generally, tissue-invasive disease in the liver of relatively immunocompromised patients is similar to that in mononucleosis-like syndrome but with more frequent jaundice and higher levels of liver enzyme elevation [13]. Rare cases of fulminant hepatic failure secondary to CMV have been reported. Of note, the reported cases involved immunocompetent patients with histologic evidence of CMV based on staining but not classic cytopathic findings [29, 30]. Histologic findings of granulomatous hepatitis have also been described in the setting of CMV infection [31, 32].

#### 3.3.3. Pancreatic

Acute pancreatitis secondary to CMV infection is well-established with confirmatory histologic evidence of infection mainly found in autopsy studies and pancreas allograft biopsies [33, 34]. An autopsy study found that approximately 10% of patients with CMV infection have pancreas involvement, although selection bias of severe disease is expected [35]. Most diagnoses in case reports are based on acute pancreatitis in the setting of CMV viremia with demonstrated response to CMV treatment [36–38]. Cholangitis with CMV involvement of the biliary epithelium has also been reported [39]. Therefore, although rare, these clinical entities merit consideration, especially in immunocompromised patients.

### 3.4. Diagnosis

An important consideration in the diagnosis of tissue-invasive disease in the general population is the assumption of immunocompetence. Even in younger patients with no reported comorbidities, a certain subset presenting with CMV colitis is soon after diagnosed with IBD [40]. Patients with prominent tissue-invasive CMV should therefore be closely evaluated for immunocompromising conditions, concurrent IBD, or an alternative diagnosis (Figure 1) [41, 42].

A diagnosis of tissue-invasive CMV requires histopathologic demonstration of CMV involvement of the suspected organ. Cytopathic effects, such as inclusion bodies, are seen in only approximately 65% of cells that have positive CMV immunohistochemical (IHC) staining [43, 44]. IHC and mucosal PCR techniques appear complimentary as approximately 10-15% of diagnoses missed by one modality can be detected by the other [45].

In relatively immunocompetent patients, the clinical utility of tissue IHC staining or PCR over cytopathic effects in the diagnosis of CMV infection has been questioned. In one center, of approximately 600 GI specimen IHC positive for CMV, none of the specimens without cytopathic effects resulted in a change in management or outcome [43]. This suggests that cytopathic changes such as inclusion bodies are the only clinically relevant histologic finding which might prompt treatment for CMV in the general population.

### 3.5. Management

Ganciclovir is an acyclic guanine nucleoside analog that is similar in structure to acyclovir but effective against CMV at concentrations 10 to 100 times lower due to an additional hydroxymethyl group [46]. Viral phosphorylation of ganciclovir occurs via UL97 during CMV replication, and subsequent phosphorylation is performed by cellular kinases to produce a competitive substrate for the CMV-DNA polymerase UL54 [47]. Mutations in the UL97 and UL54 genes are therefore the two mechanisms described for ganciclovir resistance.

Ganciclovir oral bioavailability is 6-9%, and therefore, oral ganciclovir is not recommended for treatment of CMV. The L-valyl ester, a prodrug of ganciclovir, valganciclovir, has an oral bioavailability of 61%, which improves by 25% if taken with food. Clinical trials in AIDS and solid organ transplant patients have demonstrated comparable efficacy and safety between intravenous ganciclovir and oral valganciclovir, with treatment efficacy of over 80% [48, 49].

Randomized controlled trials of prophylaxis or treatment of CMV infection in critically ill patients have not shown benefits in any clinical outcomes such as mortality or length of stay [50, 51]. No guidelines exist for treatment of tissue-invasive disease in the general population, and discretion is generally left to the treating physician based on assessed risk and benefit. As with mononucleosis-like syndrome, successful treatment with the antivirals ganciclovir, valganciclovir, and foscarnet has been reported in the literature but in the absence of a controlled study [52–54]. Severe CMV disease, such as perforation or impending liver failure, certainly warrants antiviral therapy regardless of the perceived host immune status.

## 4. CMV in IBD: Presentation, Diagnosis, and Management

CMV is found in 10-38% of patients with active ulcerative colitis (UC) based on histology and mucosal PCR technique [55, 56]. Detection of CMV in patients with active Crohn's disease (CD) is less common, presumably due to the Th1-driven pathophysiology of CD resulting in high levels of IFN-*γ* which inhibit CMV replication. TI-GI CMV is the most common manifestation of CMV disease in IBD, and given the similarity in clinical presentation with an acute IBD flare, determining the primary process clinically can be challenging.

Patients with active UC resistant to corticosteroids or multiple lines of immunosuppressive therapy are at an increased risk of clinically significant TI-GI CMV colitis. Approximately one-third of patients with steroid-refractory UC have TI-GI CMV, significantly more than steroid-responsive or inactive disease [57, 58]. Multiple cohort studies demonstrate an increased rate of histologic detection and virologic burden of CMV based on tissue IHC and DNA-PCR in patients with steroid-refractory UC compared to those with steroid-responsive disease [7, 57, 59]. Additionally, rates of surgery appear to be reduced by antiviral therapy in patients with histologic CMV and steroid-refractory disease, suggesting clinically significant TI-GI CMV [7].

On the other hand, corticosteroids as an independent risk factor for clinically significant TI-GI CMV have not been clearly established. In fact, Roblin et al. demonstrated that tissue CMV-PCR was predictive of steroid-refractory UC in a steroid-naïve patient population [60]. Other studies further suggested no association between prior steroid, immunomodulator, or biologic exposure and TI-GI CMV in patients with UC flare [27, 61]. Anti-TNF therapy has been repeatedly shown to have no discernable effect on CMV activation [62, 63]. These findings suggest that rather than being activated by immunosuppressive therapy or steroids, CMV is a primary pathogen that induces nonresponse to immunosuppressive therapy in at least a subset of patients with refractory UC and TI-GI CMV.

### 4.1. Diagnosis

Diagnosis of TI-GI CMV in IBD currently relies on histology with IHC or mucosal PCR. In the setting of endoscopic mucosal disease, 11 and 16 biopsies were required from UC and CD patients, respectively, to achieve 80% probability of CMV detection in at least one biopsy [64, 65]. By comparison, only 3 biopsies were diagnostic in 80% of AIDS patients with CMV esophagitis and visible ulcers, suggesting that a higher number of biopsies are necessary to rule out CMV in the colon or in patients with IBD or both. Endoscopic features of CMV colitis cannot be reliably distinguished from active IBD without CMV and can include erythema, exudate, erosions, and deep ulcers [66, 67].

TI-GI CMV, especially in steroid-refractory UC, occurs almost exclusively in seropositive patients [57, 60]. Therefore, CMV IgG testing can be considered as the first step of evaluation in patients with a low likelihood of latent CMV since a negative CMV IgG can preclude further testing. Other modalities of noninvasive evaluation are an unreliable surrogate for tissue-invasive CMV in patients with IBD. Between 33-50% of patients with biopsy-confirmed CMV colitis, including high tissue disease burden, can have negative serum PCR or antigenemia. Similarly, low-level antigenemia or serum PCR can be common especially in the setting of steroid or cyclosporine immunosuppression and generally self-resolves [68]. On the other hand, a high serum CMV viral load can be suggestive of steroid refractoriness in active UC [65, 69]. Therefore, the absence of detectable serum CMV should not preclude further evaluation for CMV colitis; however, high serum levels may encourage treatment especially if invasive testing is being deferred [65, 68, 69].

Small studies of stool-based PCR testing in patients with IBD have demonstrated a sensitivity and specificity of 67-83% and 93-96%, respectively, compared to PCR of colonic mucosal biopsies [70, 71]. However, the clinical utility of this test is less certain.

Disease location is an important consideration when planning for endoscopy. In a study by McCurdy et al., TI-GI CMV disease was seen exclusively proximal to the splenic flexure in 50% of CD and only 9% of UC patients [65, 72]. Therefore, whereas flexible sigmoidoscopy may suffice in most cases for UC, a colonoscopy may be necessary to sufficiently rule out CMV of the colon in other IBD patient populations.

### 4.2. Management

Treatment of CMV in patients with IBD should be reserved to patients where TI-GI CMV is felt to be a significant driver of GI inflammation. CMV as a pathogen in the setting of active IBD has been demonstrated in patients where antiviral therapy and reduction of immunosuppression have induced significant clinical improvement [34, 73]. Untreated CMV infection in IBD is generally associated with increased risk of hospitalization, colectomy, and mortality compared to IBD patients without active CMV [59, 74–77]. Studies that fail to demonstrate an effect of CMV infection on IBD outcomes may be accounted for by variances in the CMV burden, where reactivations associated with lower CMV burden are less likely to result in clinically significant CMV-driven disease [59, 68]. Since CMV infection in patients with IBD is associated with rare IHC staining in approximately 50% of histologic specimen, most cases of CMV in gastrointestinal tissue are likely reactivation due to local immune dysregulation that has no or minimal clinical consequences [43, 65, 78].

**Figure 2:**
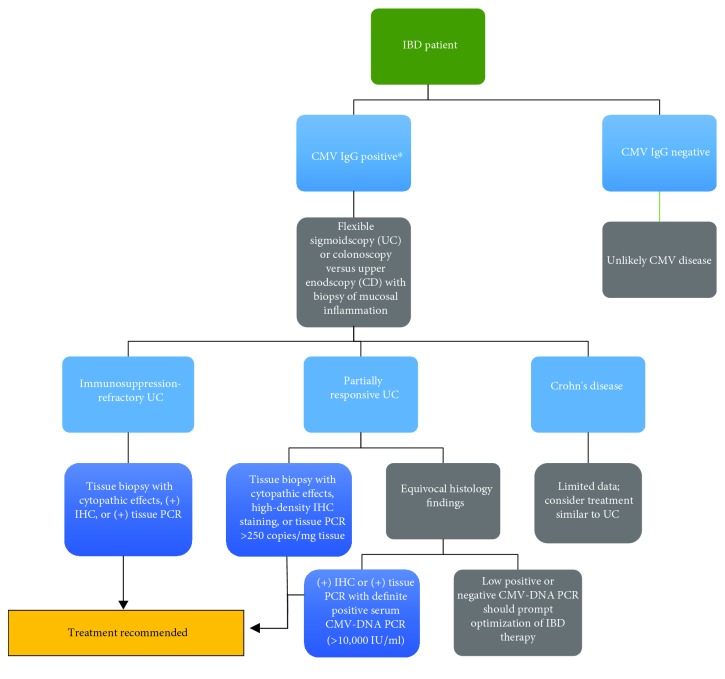
Flow diagram for diagnostic approach in patients with suspected CMV (continued). ^∗^Not required. Should not delay endoscopic evaluation per routine care for active colitis.

High CMV tissue burden on the other hand has been shown to correlate with steroid-refractory IBD and response to antiviral therapy [59, 79]. Roblin et al. demonstrated that a CMV PCR greater than 250 copies per mg of colon tissue was associated with resistance to three successive treatment regimens to UC, and 88% of these patients improved with intravenous ganciclovir [60]. A case-control study by Jones et al. demonstrated that antiviral therapy in IBD patients with high-grade CMV disease improved surgery-free survival especially when compared to low-grade CMV disease burden [79]. In case series and case-control studies of steroid-refractory UC, patients with positive IHC staining or tissue PCR, even in the absence of cytopathic changes, tend to respond to antiviral treatment with significantly lower rates of surgery or surgery-free survival [7, 57, 67]. Therefore, antiviral therapy in refractory UC with histologic evidence of CMV or UC patients with high burden of tissue CMV should be strongly considered (Figure 2).

Antiviral therapy in the setting of IBD as mentioned above is similar to the general population. Experience in the IBD community generally involves a 2-3-week course of antiviral therapy with intravenous ganciclovir 5 mg/kg twice daily for 5-10 days followed by valganciclovir 900 mg daily for the remainder of the course [57, 60, 80]. An earlier transition to oral valganciclovir after 3-5 days of intravenous ganciclovir may be reasonable with early responders. Immunosuppression reduction, especially corticosteroids, azathioprine, and cyclosporine should be strongly considered in patients with high suspicion for TI-GI CMV based on increased risk of CMV reactivation with these medications in the solid organ transplant and IBD populations [68, 81]. Depending on the clinical course, both antiviral and immunosuppression may be required to achieve clinical remission. In such a case, an anti-TNF agent would be preferred for the reasons mentioned previously.

Finally, intensive granulocyte and monocyte adsorptive apheresis (GMAA) twice a week has been shown equally effective in inducing clinical remission of active UC in patients with and without colonic TI-GI CMV based on IHC and PCR [82]. In fact, histologic resolution of CMV occurred in 73.3% of affected patients with intensive GMAA, compared with 87.5-100% histologic clearance with antiviral therapy [57, 60, 82]. GMAA however has several limitations. Efficacy has been best demonstrated in moderate-severe ulcerative colitis, patients without severe ulceration, and steroid-naïve patients. Even then, a randomized, double-blinded, sham-controlled study in the United States, Europe, and Japan failed to demonstrate benefit in moderate-severe ulcerative colitis [83]. Therefore, experience, availability, and insurance coverage in the United States are limited.

## 5. CMV in Liver Transplant: Presentation, Diagnosis, and Management

CMV infection is one of the most common opportunistic infections in patients following solid organ transplant (SOT) and occurs in up to 55% of post-LT patients [84]. Risk of infection is driven by a variety of factors including host comorbidities, posttransplant immunosuppressive protocol, allograft rejection, and most importantly patient and donor pretransplant CMV seropositivity [85]. A seronegative recipient and seropositive donor (R-/D+) match confers the highest risk for CMV infection with rates of 78-88% without prophylaxis, whereas D-/R- status confers the lowest risk and occurs in 0-13% of patients (Table 1) [84, 86].

CMV syndrome constitutes approximately 60% of CMV disease in LT patients and is characterized by a combination of constitutional, nonlocalizable symptoms, hematologic dyscrasias, and liver enzyme elevation [87]. Tissue-invasive disease most frequently involves the liver allograft due to aberrant local immune response, with detectable CMV in the allograft of 11-17% of post-LT patients. The gastrointestinal tract is the next most common site with clinical presentations similar to TI-GI CMV in other patient populations [86, 88].

The effects of CMV on allograft function extend beyond direct infection, however, as risks of acute and chronic allograft rejection are increased in patients with CMV infection [85]. Risks of bacteremia, invasive fungal disease, EBV-associated posttransplant lymphoproliferative disease, and cardiovascular disease are also increased in these patients [85, 89]. Therefore, despite the efficacy of treatment of CMV disease or infection with antiretrovirals, prevention is a major strategy in post-LT patients.

### 5.1. Diagnosis

Serology plays an important role pretransplant in detection of latent infection given implications on posttransplant risks of CMV infection and disease. While acute CMV infection can also be detected using IgM antibody or IgG antibody paired samples, the sensitivity of these tests in SOT patients is almost 50% at the time of symptom onset. By comparison, antigenemia and PCR have sensitivities of 79-80% and 84-100% in this setting, respectively [90–93]. These observations are explained by narrow detection windows and reliance of serology on an intact immune system [47, 93, 94].

While diagnostic performance of antigenemia is generally comparable to PCR in the serum, its main limitations are reliance on an adequate neutrophil count, operator dependence, and the need to process samples within 6-8 hours. For all the reasons above, nucleic acid detection with serum PCR is the preferred test for real-time detection of CMV infection in LT patients [47].

A precise diagnosis of probable CMV syndrome can be made using criteria established by a panel of experts from the CMV Drug Development Forum which is based on demonstration of CMV viremia in addition to other clinical parameters that include liver enzyme elevation, thrombocytopenia, leukopenia, and presence of fatigue, malaise, or fever [11].

Diagnosing tissue-invasive disease of the allograft does not require histology and can be made based on CMV viremia, liver enzyme elevations, and exclusion of alternative causes of hepatitis. However, since CMV infection is a risk factor for allograft rejection and vice versa, a liver biopsy is generally performed to distinguish between these two processes [47].

Definite TI-GI is based on endoscopic and histologic evidence of CMV disease. Unlike the AIDS and IBD populations, no studies have examined the location of colonic TI-GI CMV in SOT patients. Since isolated ileal involvement with TI-GI CMV has been reported in posttransplant patients, initial investigation of suspected lower GI tract CMV disease with colonoscopy and ileal intubation would be prudent in this population until more data becomes available [95, 96].

### 5.2. Management

Universal prophylaxis for a minimum of 90 days is currently recommended based on the relatively higher degree of immunosuppression during this time period. Clinical trial data has shown up to 80% reduction of CMV infection with prophylaxis during the first 90 days after transplant [97]. In lower-risk patients, multiple randomized clinical trials in renal transplant patients have demonstrated comparable efficacy between universal prophylaxis and a strategy of preemptive therapy with weekly monitoring of serum CMV. A meta-analysis of retrospective studies in post-LT patients has largely supported this conclusion but suggested decreased graft loss with universal prophylaxis [98]. Results from a randomized clinical trial in LT patients are expected soon (NCT01552369).

Treatment for CMV should be initiated for CMV disease or increasing viremia in asymptomatic CMV infection (Figure 1). Generally, a greater than threefold increase in serum CMV PCR is considered significant [48, 99]. A large, multicenter, noninferiority trial of SOT patients did not demonstrate inferiority of oral valganciclovir to intravenous ganciclovir in viremia eradication or treatment success and showed comparable safety [49]. First-line treatment of CMV infection is therefore IV ganciclovir 5 mg/kg twice daily or valganciclovir 900 mg twice daily adjusted for renal impairment, and efficacy of treatment is up to 85% in immunocompromised HIV/AIDS and postorgan transplant patients [46, 49].

Even after successful treatment of CMV disease, relapse rates can be as high as 27%. Persistent viremia based on CMV PCR at completion of a 14-day course was associated with relapse; therefore, treatment until undetectable levels is recommended and can be continued until multiple negative levels are obtained, generally at least 2 weeks apart [47, 100]. High CMV burden as evidenced by either viremia or severe tissue-invasive disease increases risk of relapse; however, endoscopic resolution of TI-GI disease was not predictive of relapse risk [101]. Secondary prophylaxis does not appear to confer a protective effect on relapse risk beyond delaying time to relapse while on treatment [102].

Another important consideration in the treatment of CMV is the level of immunosuppression, as generally decreasing immunosuppression will help with treatment. Use of mTOR appears to be associated with decreased incidence of CMV infection [103].

The incidence of ganciclovir-resistant CMV in LT patients is not well-defined but is relatively rare in SOT patients, ranging from 2% overall to 7% in high-risk R-/D+ patients [104, 105]. Testing for resistance is available and can help guide treatment in nonresponders to first-line agents [106]. UL97 mutations will render ganciclovir and valganciclovir ineffective and can be circumvented with foscarnet and cidofovir. UL54 mutations are significantly more challenging and are generally approached with combination therapy involving first-line, second-line, and experimental treatments [106].

## 6. Summary

CMV infection and disease are frequently encountered entities in the practice of gastroenterology and hepatology. Direct detection via PCR and IHC has drastically improved our ability to detect CMV in host tissue; however, determining true CMV disease remains a clinical diagnosis and challenging in certain populations. Treatment decisions should therefore be based on a combination of factors assessing host immune status and viral burden. Valganciclovir is an effective treatment option for CMV infection in all patient populations and has significant impact on graft and patient survival in post-LT patients through prophylaxis and treatment.

## Figures and Tables

**Table 1:** Definitions and risk factors of the described CMV clinical entities in the general, IBD, and liver transplant patient populations.

Disease manifestation	Definition	Risk factors
*General population*		
Latent CMV	Asymptomatic, no clinically detectable active replication	Female sex, older age, high crowding index, and low household income or education [2]
Mononucleosis-like syndrome	Predominant constitutional, mononucleosis-like symptoms with intact immune system	Blood transfusions, second or third decades of life, exposure to bodily fluids of infected host [14]
Tissue-invasive CMV	Predominant symptoms localizable to a specific tissue site	Critical illness (especially with sepsis or intubation at time of admission), active malignancy (especially with low BMI, lymphopenia, or steroid use), hematologic malignancy, blood transfusion [27]
*Inflammatory bowel disease* ^∗^		
Tissue-invasive CMV	Predominant symptoms typically localizable to the GI tract, mimicking IBD flare	Endoscopic inflammation [107], immunosuppressant-refractory ulcerative colitis
*Liver transplant* ^∗∗^		
CMV syndrome	Positive CMV serum PCR with 2 of the following: (i) Fever ≥ 38°C for at least 2 days (ii) New fatigue or malaise (iii) Leukopenia or neutropenia (iv) ≥5% atypical lymphocytes (v) Platelets < 100,000 cell/*μ*L or >20% decrease (vi) >2 xULN ALT or AST	R-/D+ status, acute allograft rejection, immunosuppression with antilymphocyte antibodies, mycophenolate dose > 2 grams/day, viral coinfection, toll-like receptor polymorphisms [108]
Tissue-invasive CMV	Predominant symptoms localizable to a specific tissue site	

^∗^Latent CMV and mononucleosis-like syndrome definition and risk factors are as with the general population. ^∗∗^Latent CMV definition and risk factors are as with the general population.
